# Growth performance, antioxidant, and immune responses of Nile tilapia (*Oreochromis niloticus*) fed on low-fishmeal diets enriched with sodium chloride and its adaptability to different salinity levels

**DOI:** 10.1007/s10695-024-01426-2

**Published:** 2025-01-09

**Authors:** Mohamed N. Monier, Asmaa S. Abd El-Naby, Reham M. Fawzy, Fatma Samir, Sherien H. H. Shady, Youssif Shehata Grana, Najah M. Albaqami, Mohsen Abdel-Tawwab

**Affiliations:** 1 Department of Fish Biology and Ecology, Central Laboratory for Aquaculture Research, Agricultural Research Center, Abbassa, Abo-Hammad, 44662 Sharqia Egypt; 2 Department of Fish Nutrition, Central Laboratory for Aquaculture Research, Agricultural Research Center, Abbassa, Abo-Hammad, 44662 Sharqia Egypt; 3 Limnology Department, Central Laboratory for Aquaculture Research, Agricultural Research Center, Abbassa, Abo-Hammad, Sharqia Egypt; 4 Department of Biological Sciences, Faculty of Sciences, King Abdulaziz University, Jeddah, Saudi Arabia

**Keywords:** Dietary NaCl, Nile tilapia, Growth performance, Welfare status, Salinity stress

## Abstract

The current investigation assessed the beneficial impacts of dietary sodium chloride (NaCl) on the growth performance, oxidant/antioxidant, and immune responses of Nile tilapia (*Oreochromis niloticus*) and its adaptability to different salinity levels. After acclimating the fish to the laboratory conditions for 2 weeks, the acclimated fish (10.5 ± 0.16 g) were randomly distributed into 25 110-L rectangular glass tanks (15 fish/tank) to represent five groups in five replicates. The fish were fed with experimental feeds fortified with 0.0 (control), 5, 10, 15, and 20 g NaCl/kg feed for 60 days. Following the nutritional experiment, fish of all groups were adapted to different salinity levels from 0 to 32 g /L for a further 3 weeks, during which fish mortality was recorded. Blood samples were taken after the feeding trial and at a salinity level of 24 g/L. Growth performance and hematological parameters (WBCs, RBCs, hemoglobin, and hematocrit), total protein, albumin, globulin, digestive enzymes, antioxidant activity, and immunity status were markedly improved with increased NaCl rates in the fish diets up to 10 g/kg feed, after which all previous parameters were declined. On the other hand, feeding fish on a diet containing 10 g NaCl/kg feed showed substantially lower levels of cortisol, glucose, cholesterol, triglycerides, aspartate transaminase (AST), alanine transaminase (ALT), and malondialdehyde (MDA). Exposing the control fish group to salinity stress (32 g/L) for 3 weeks markedly decreased their digestive enzyme activity, immunity status, and antioxidant response, along with significant increases in cortisol, glucose, cholesterol, triglycerides, AST, ALT, and MDA levels. Conversely, feeding fish on a diet containing 10 g NaCl/kg feed alleviated the negative impacts of salinity stress and helped fish to tolerate salinity stress up to 24 g/L.

## Introduction

The most important input for successful aquaculture is the feed, which mainly depends on fishmeal as a main protein source. Fishmeal is a typical protein source in aqua-feeds due to its high nutritive value, high digestibility, and great palatability, along with minimum anti-nutritional factors (Gatlin et al. 2007; Hardy 2010; Tacon et al. 2011; Oliva-Teles et al. 2015). Because of the gradual increase in fishmeal use in aqua-feeds as well as other animals’ feeds, the global demand will soon outpace the production, driving up the cost of fishmeal. Hence, many plant proteins (PP) were used to substitute fishmeal protein in freshwater and marine fish diets due to their availability and inexpensive cost (Kaushik and Hemre 2008; Hardy 2010; Tacon et al. 2011; Oliva-Teles et al. 2015; Saleh et al. 2021a, b; Tewari 2022; Akter et al. 2023). Most PP sources contain anti-nutritional factors, which negatively affect the palatability of the feed, leading to low feed consumption, lowering the fish growth, and deteriorating the fish health status (Glencross et al. 2020; Aragão et al. 2022). Furthermore, PP-rich sources may cause to undergo cellular and morphological changes in fish gut histo-morphometry, impairing their ability to digest and absorb nutrients (Wang et al. 2017; Tran-Ngoc et al. 2019), leading to subsequent deterioration in fish growth and welfare status (Saleh et al. 2021a, b).

Fishmeal is rich in sodium (Na), while PP sources are often poor in Na. As a result, substituting fishmeal with PP-based sources in fish diets may lead to Na deficiency (Nakajima and Sugiura 2016). Adding NaCl to low-fishmeal diets considerably affects their growth, feed consumption, and physiological variables (Cnaani et al. 2010; Nakajima and Sugiura 2016). Sodium plays a significant role in the intestinal uptake of multiple nutritional elements, which is assisted by various Na + -dependent carriers (Ganapathy and Leibach 1985; Ferraris 2001; Bakke et al. 2010). Moreover, from an economic standpoint, including NaCl in commercial feeds may be beneficial as it diminishes the quantity of other components (nutrient dilution) in proportion to the quantity of NaCl applied (Harpaz et al. 2005).

Nile tilapia (*Oreochromis niloticus*) is one of the most extensively farmed freshwater fish species that was distributed from Africa to so many countries worldwide due to its tolerance to many environmental conditions and well-confirmed rearing way (El-Sayed and Fitzsimmons 2023). Globally, most tilapia farming is carried out in freshwater facilities with a salinity range of 10–12 ppt without any detrimental effects on their growth performance, immune response, and stress resistance of the fish (Dawood et al. 2023). Due to the limited availability of freshwater in some regions, brackish and sea waters might be used as alternatives to fresh water in the Nile tilapia farms (El-Sayed 2006; Dawood 2021). However, the gradual acclimation of Nile tilapia to seawater can be successful. In this case, this would open many tropical and arid coastal areas to tilapia production and could significantly expand the global production of this important tilapia species.

Freshwater fish are hyperosmotic to their ecological surroundings and exhibit physiological issues associated with solute loss. Furthermore, these fish compensate for this loss by actively absorbing salt ions from the environment (Tavares-Dias 2022). Additionally, fish may satisfy their osmoregulatory demands via diets with high levels of sodium chloride (NaCl) (Gatlin et al. 1992; Gangadhara et al. 2004; Tavares-Dias 2022). However, the NaCl quantity required for the development and growth of freshwater fish should be considered. Hence, this study has been planned to evaluate the beneficial impacts of dietary NaCl on the growth performance, hemato-biochemical, immune, and antioxidative biomarkers of Nile tilapia and its adaptability to different salinity levels.

## Materials and methods

### Diets preparation and fish management

Dietary NaCl was obtained from a local market in Zagazig, Egypt, and enriched to a control diet (30% crude protein; CP) at a rate of 0.0 (the control), 5, 10, 15, and 20 g/kg feed (Table 1). The diet’s contents were well-stirred for 30 min, during which 200 mL of water was added to every 1-kg feed. The feed pastes underwent processing in a meat grinder, while the diet threads were air-dried at ambient temperature prior to being crushed to a diameter of 2 mm. The experimental feeds were stored at − 4.0 °C, pending they were utilized.

**Table 1:** Ingredients and proximate chemical analysis (%; on dry matter basis) of diets containing different levels of sodium chloride (NaCl)

Ingredients	NaCl levels (g/kg diet)				
	0.0 (Control)	5	10	15	20
Fish meal (65% CP)	85	85	85	85	85
Soybean meal (48% CP)	230	230	230	230	230
Corn gluten (63% CP)	110	110	110	110	110
Corn (8.3% CP)	130	130	130	130	130
Defatted rice bran (21.7 CP)	180	180	180	180	180
Wheat mill run	185	185	185	185	185
Soybean oil	20	20	20	20	20
Vitamins premix ^a^	10	10	10	10	10
Minerals premix ^b^	10	10	10	10	10
Calcium diphosphate	5	5	5	5	5
Starch	35	30	25	20	15
NaCl	0	5	10	15	20
Total	1000	1000	1000	1000	1000
Chemical composition					
Dry matter	89.39	89.42	89.46	89.50	89.53
Crude protein	32.12	32.12	32.12	32.12	32.12
Total lipids	5.98	5.98	5.98	5.98	5.98
Crude fiber	4.53	4.52	4.52	4.51	4.50
Total ash	7.19	7.64	8.08	8.53	8.98
Nitrogen-free extract ^c^	50.17	49.73	49.92	48.85	48.41
GE (kcal/100 g) ^d^	460.43	458.59	456.75	454.91	453.10

^a^Vitamin mixture (IU or mg kg^−1^ diet): DL-alpha tocopherol acetate. 60 IU; DL-cholecalciferol, 3000 IU; thiamin, 15 mg; ribofelavin, 30 mg; pyridoxine, 15 mg; B 12, 0.05 mg; nicotinic acid, 175 mg; folic acid, 5 mg; ascorbic acid, 500 mg; inositol, 1000 mg; biotin, 2.5 mg; calcium panthoteate, 50 mg; choline chloride, 2000 mg

^b^Mineral mixture (g or mg kg^−1^ diet): calcium carbonate (40% Ca), 2.15 g; magnesium oxide (60% Mg), 1.24 g; ferric citrate, 0.2 g; potassium iodide (75% I), 0.4 mg; zinc sulfate (36% Zn), 0.4 g; copper sulfate (25% Cu), 0.3 g; manganese sulfate (33% Min), 0.3 g; dibasic calcium phosphate (20% Ca, 18% p), 5 g; cobalt sulfate, 2 mg; sodium selenite (30% Se), 3 mg; potassium chloride, 0.9 g; sodium chloride, 0.4 g

^c^Nitrogen-free extract (calculated by difference) = 100 – (protein% + lipid% + ash% + fiber%)

^d^GE was calculated according to NRC (2011) in Kcal/100 g = (crude protein × 5.6) + (crude lipid × 9.44) + [4.1 × (crude fiber + NFE)]

Nile tilapia (*O. niloticus*) juveniles were procured from the breeding ponds at World Fish at Abbassa, Abo-Hammad, Sharqia, Egypt, and adapted to indoor lab environments for 14 days, while providing the commercial diet (30% CP). After fish acclimation, healthy fish (10.5 ± 0.16 g) were randomly spread into 25 110-L rectangular glass tanks (15 fish/glass tank) to represent five groups with five replicates. The glass tanks were filled with dechlorinated tap water and aerated with an air compressor via air stones. The experimental feeds were given to the fish three times a day until they reached a state of apparent satiety at 9:30, 12:30, and 15:30 h for 60 days. Biweekly, all tank’s fish were gathered and weighed together to evaluate fish growth. Each day, around 50% of the tanks’ water, along with fish waste, was siphoned and partly substituted with new well-aerated dechlorinated tap water, which was supplied from a storage tank.

Water temperature and dissolved oxygen were tested daily in sites by a transportable oxygen meter (Jenway, London, UK). A pH meter (Digital Mini-pH Meter, model 55, Fisher Scientific, Denver, CO, USA) was utilized to test the pH degree. The HACH comparison equipment was used to determine the unionized ammonia concentration, according to Boyd (1984). The following parameters were recorded: (i) the water temperature was about 25.3 to 27.4 °C; (ii) the dissolved oxygen concentration was between 5.4 and 5.7 mg/L; (iii) the pH was between 7.4 and 7.7; and (iii) the unionized ammonia concentration was between 0.023 and 0.025 mg/L. All water parameters were appropriate for the tilapia culture (Boyd and Tucker 2012).

Subsequent to the experimental trial (60 days), fish in all glass tanks were starved for 24 h prior to sampling and anesthetized with tricaine methanesulfonate (MS 222; 30 mg/L) for blood sampling, and fish weighed. All fish were harvested, enumerated, and group-weighed, and their performance indices were determined as the following equations:

$$\begin{array}{l}\mathrm{Weight}\;\mathrm{gain}\;\%=100\lbrack\mathrm{final}\;\mathrm{weight}\;\left(\mathrm{FW}\right)-\mathrm{initial}\;\mathrm{weight}\;(\mathrm{IW})\rbrack/\mathrm{initial}\;\mathrm{weight}\;(\mathrm{IW});\\\mathrm{Specific}\;\mathrm{growth}\;\mathrm{rate}\;(\mathrm{SGR};\%/\mathrm{day})=100\lbrack\mathrm{Ln}\;\mathrm{FW}\;\left(\mathrm g\right)-\mathrm{Ln}\;\mathrm{IW}\;(\mathrm g)\rbrack/60;\\\mathrm{Feed}\;\mathrm{conversion}\;\mathrm{ratio}\;\left(\mathrm{FCR}\right)=\mathrm{total}\;\mathrm{dry}\;\mathrm{feed}\;\mathrm{intake}/\mathrm{weight}\;\mathrm{gain};\\\mathrm{Fish}\;\mathrm{survival}\;\left(\%\right)=100(\mathrm{fish}\;\mathrm{number}\;\mathrm{at}\;\mathrm{the}\;\mathrm{trial}\;\mathrm{end}/\mathrm{fish}\;\mathrm{number}\;\mathrm{at}\;\mathrm{the}\;\mathrm{trial}\;\mathrm{beginning}).\end{array}$$

### Salinity adaptability

Following the nutritional experiment, fish per each treatment were grouped and randomly redistributed into ten 110-L glass tanks, each with twenty fish representing five treatments with two replicates. The fish were nourished with the same diets to represent the same treatments. Fish were acclimated to several salt levels (6, 12, 15, 18, 20, 24, and 32 g/L), with a progressive rise every 2 days, and kept up to the third week (the total period of salinity adaptability was 3 weeks). Fish deaths in each glass tank were documented every day. Then, three fish from each glass tank were sampled for further blood specimens and intestine and liver samples at a salinity level of 24 g/L.

### Blood and fish sampling

After the feeding trial, blood specimens were collected from three fish per tank (15 fish/treatment) with a sterilized syringe (containing 500 U sodium heparin) from the caudal vein and split into two groups of Eppendorf tubes. The first group was used to determine the hematological variables, phagocytic activity, and respiratory burst activity. While the second group was centrifuged at 6000 × g for 15 min at ambient temperature, and the gotten plasma was kept at − 20 °C for additional biochemical analyses.

Following the collection of blood, the sedated fish were euthanized using the medullary section and then dissected. Mid-parts of the fish intestine and liver tissues were gathered after the feeding trial and during salinity stress at salinity 24 g/L and reserved at − 18 °C to evaluate mid-intestinal digestive enzymes and hepatic antioxidative parameters.

### Digestive enzyme measurements

The mid part of intestinal tissues was homogenized in 0.9% physiological saline solution (1:9) and was subjected to centrifugation at a temperature of 4 °C for 15 min at a speed of 5000 revolutions per minute. The supernatants were assembled in a sterile manner and stored at a temperature of − 20 °C until they were ready for further analysis. Following Bernfeld (1955), α-amylase activity was measured utilizing 1% starch solution in 0.1 M Tris–HCl buffer (pH 7.0) as a substrate. Protease activity was determined by digesting 0.1 mL crude enzyme extract with 2.0 mL casein buffer substrate at 28 °C for 15 min and measuring the supernatant absorbance at 280 nm (Kunitz 1947). Following Cherry and Crandall (1932), lipase activity was measured by hydrolyzing triglyceride in olive oil. Total protein (TP) was quantified using Henry (1964) techniques, and enzyme activity was reported as U/mg protein.

### Hemato-biochemical assays

The methods of Brown (1980) were used for blood analyses. An optical microscope (× 400 magnification) and Neubauer hemocytometer were used to count red blood cells (RBCs) and white blood cells (WBCs) in blood samples. Hemoglobin (Hb) levels were determined using commercial kits (Biodiagnostic Co., Cairo, Egypt) at 540 nm. Hematocrit was calculated by centrifuging blood (5 min, 1400 g) in heparinized glass capillaries. Mean corpuscular volume (MCV, fl), MCH (pg), and MHC (g/dL) were calculated using these blood parameters.

Commercial ELISA packets (Shanghai Enzyme Biotechnology Co., Ltd.) were used to assess blood cortisol levels before and after the salinity challenge (Foster and Dunn 1974). Blood glucose, total cholesterol (T-CHO), triglyceride (TG), aspartate aminotransferase (AST), and alanine aminotransferase (ALT) levels were assessed before and after the salinity stress using an automated biochemical analyzer (ADVIA 2400; SIEMENS; Hamed and Abdel-Tawwab 2021).

### Immune assays

By means of diagnostic kits (Biodiagnostic Co., Cairo, Egypt), serum biochemical assays were assessed both prior to and after the salinity challenge, as described by the manufacturers’ guidelines. The measurements of total serum protein (TP) and albumin (ALB) were determined as outlined in the protocols established by Henry (1964) and Doumas et al. (1971), respectively. In order to determine globulin (GLOB) levels, ALB values were subtracted from TP values. The activities of lysozyme (LYS) were assessed before and after the salinity challenge using *Micrococcus luteus* as a substrate (Ellis 1990). A unit of LYS activity was established as the count of enzymes responsible for inducing a 0.001 OD/min decrease in the absorbance of 1 mL of serum. Total immunoglobulin (total Ig) was quantified using the protocol outlined by Siwicki and Anderson (1993). According to Secombes (1990), the respiratory burst (RB) activity was assessed in whole fresh blood using the nitro-blue-tetrazolium assay from diagnostic reagent kits (Randox, London, UK).

Following the technique mentioned by Kawahara et al. (1991), the phagocytic activity was evaluated both before and during the salinity challenge. Blood (0.1 mL) was pipetted into a coverslip and thoroughly mixed with 25 mL of *Candida* sp. The specimen was subsequently kept at ambient temperature for 2 h. In addition, it underwent a 5-min fixation process using 100% methanol, followed by a 15-min staining period with 10% Giemsa (Sigma-Aldrich, USA). Following rinsing the excessive stain with phosphate buffer solution (pH 7.4), a microscopic count of phagocyte cells per 300 adhered cells was performed. The phagocytic activity was subsequently calculated as the percentage of phagocytic cells that exhibited phagocytic activity.

### Antioxidant and lipid peroxidation assays

Liver samples underwent multiple washes in a cool 0.85% NaCl solution before being homogenized via a glass homogenizer in the physiological solution 0.9% (1 g: 9 mL). Following centrifugation at 5000 × g for 20 min, the homogenate obtained was utilized to analyze antioxidant biomarkers with diagnostic kits (Biodiagnostic Co., Cairo, Egypt). Superoxide dismutase (SOD) activity was quantified at a wavelength of 550 nm (McCord and Fridovich 1969). By measuring the H_2_O_2_ reaction at a wavelength of 240 nm, the catalase (CAT) activity was measured (Aebi 1984). The activity of glutathione peroxidase (GPx) was assessed using the methyl catechol reaction at a wavelength of 340 nm (Paglia and Valentine 1967). Malondialdehyde (MDA) was determined as an indicator of lipid peroxidation using the thiobarbituric acid reaction at a wavelength of 532 nm (Ohkawa et al. 1979).

### Statistical analysis

Prior to the statistical analysis, the data obtained were tested for the normality of distribution and homogeneity of variances among different treatments using the Kolmogorov–Smirnov test and Bartlett’s test, respectively. The data obtained for fish growth and hematological indices were analyzed using one-way ANOVA. In order to assess the interaction between dietary NaCl and salinity stress, the obtained data on blood biochemical, digestive enzyme, antioxidant response, and immunological parameters were subjected to a two-way ANOVA test. The differences among means varied significantly (*P* < 0.05) using the Tukey Test, which was used as a post-hoc test. All statistical analyses were conducted utilizing version 26 of the SPSS program (Dytham 2011).

## Results

### Growth performance

**Fig. 1:**
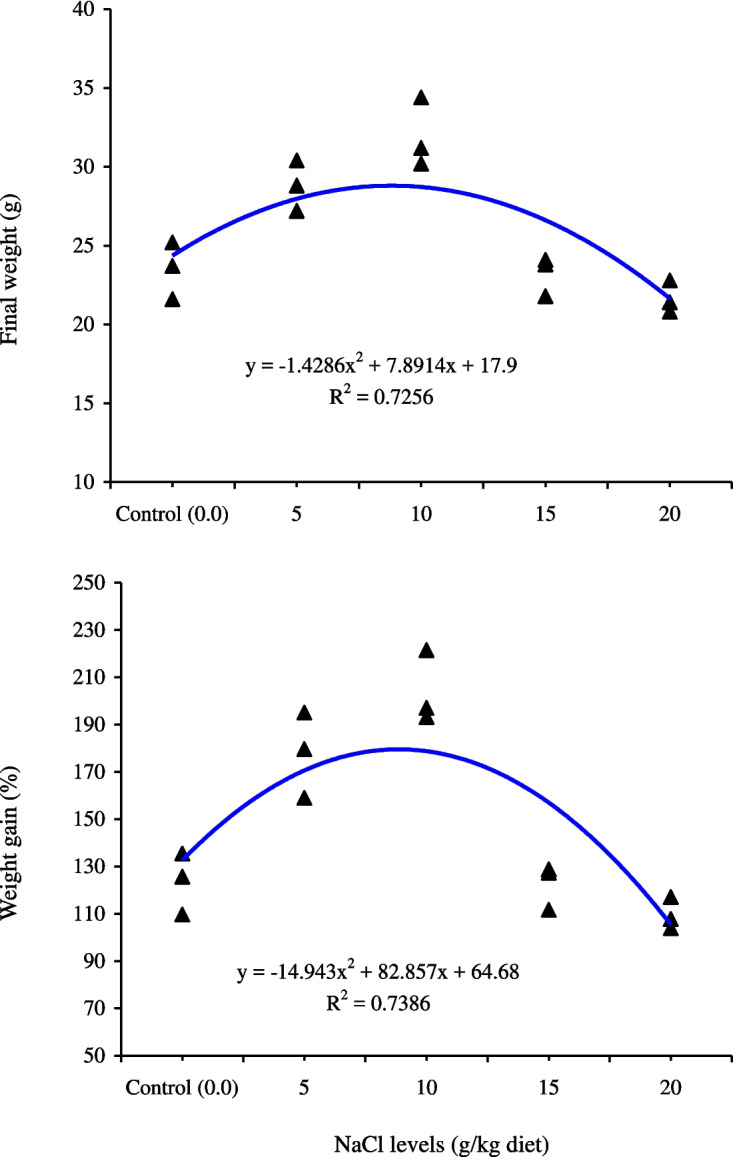
The relationship between final weight (g) and weight gain % of Nile tilapia (*O. niloticus*) fed on different levels of sodium chloride (NaCl) for 60 days

All benchmarks related to growth performance were improved significantly (*P* < 0.05) with the rise of NaCl levels in the diets up to 10 g/kg feed in quadratic trends (Table 2). However, beyond this point (10 g NaCl/kg feed), the growth performance declined. The FCR values were quadratically (*P* < 0.05) improved with NaCl feeding compared with the control group. No significant (*P* > 0.05) differences in FCR values were detected among salt-fed fish groups, and the lowest FCR value was at 10 g NaCl/kg feed (Table 2). There were no noteworthy (*P* > 0.05) variances in survival rate, and its range was 97.8–100% (Table 2). Polynomial regression showed the relationship among final weight and weight gain % against dietary sodium chloride, displaying that the optimum sodium chloride level is suggested to be 7.5–10 g/kg feed (Fig. 1).

**Table 2:** Growth performance and feed utilization indices of Nile tilapia (*O. niloticus*) fed on different levels of sodium chloride (NaCl) for 60 days

NaCl levels (g/kg feed)	Initial weight (g)	Final weight (g)	Weight gain % (%)	SGR (%/day)	Feed intake (g feed/fish)	FCR	Survival (%)
0.00	10.5 ± 0.12	23.5 ± 1.04 c	123.8 ± 7.52 c	1.34 ± 0.06 b	22.43 ± 1.07 b	1.73 ± 0.06 a	100.00 ± ± 0.00
5.0	10.4 ± 0.07	28.8 ± 0.92 b	177.8 ± 10.46 b	1.70 ± 0.06 a	28.07 ± 1.72 a	1.53 ± 0.05 b	100.00 ± 0.00
10.0	10.5 ± 0.12	31.9 ± 1.27 a	204.1 ± 8.86 a	1.85 ± 0.05 a	30.87 ± 0.87 a	1.44 ± 0.05 b	97.8 ± 2.23
15.0	10.4 ± 0.06	23.2 ± 0.72 c	122.7 ± 5.48 c	1.34 ± 0.04 b	19.87 ± 0.77 bc	1.55 ± 0.03 b	100.00 ± 0.00
20.0	10.3 ± 0.09	21.7 ± 0.59 c	109.7 ± 3.92 c	1.24 ± 0.03 b	18.17 ± 1.04 c	1.61 ± 0.05 ab	97.8 ± 2.23
*P* value							
Linear trend	0.473	0.248	0.264	0.233	0.082	0.316	0.317
Quadratic trend	0.720	0.002	0.001	0.001	0.002	0.005	0.619

Mean ± standard error values followed by different letters in the same column are significantly different at *P* < 0.05

### Hematological variables

With respect to the control treatment, WBCs, RBCs, Hct, and Hb exhibited quadratic (*P* < 0.05) increases with the rise of NaCl levels in the diets up to 10 g NaCl/kg feed, after which (15 and 20 g NaCl/kg feed) negatively impacted these parameters (Table 3). Dietary NaCl levels did not significantly (*P* > 0.05) impact MCV, MCH, and MCHC values (Table 3).

**Table 3:** Changes in hematological indices of Nile tilapia (*O. niloticus*) fed on different levels of sodium chloride (NaCl) for 60 days

NaCl levels (g/kg feed)	WBCs (× 10^3^/mm^3^)	RBCs (× 10^6^/mm^3^)	Hematocrit (%)	Hemoglobin (g/dL)	MCV (fL)	MCH (pg)	MCHC (g/dL)
0.00	9.62 ± 0.14 c	3.06 ± 0.13 c	30.00 ± 1.15 e	9.27 ± 0.36 d	98.08 ± 0.49	30.29 ± 0.16	30.88 ± 0.00
5.0	10.82 ± 0.39 bc	4.01 ± 0.05 b	39.00 ± 0.58 c	12.03 ± 0.37 c	97.25 ± 0.18	29.99 ± 0.53	30.83 ± 0.49
10.0	14.99 ± 0.78 a	4.88 ± 0.13 a	47.50 ± 1.44 a	14.30 ± 0.35 a	97.32 ± 0.31	29.30 ± 0.08	30.10 ± 0.18
15.0	10.34 ± 0.08 c	4.50 ± 0.11 a	43.00 ± 0.58 b	13.28 ± 0.22 b	95.61 ± 1.05	29.52 ± 0.23	30.88 ± 0.10
20.0	11.79 ± 0.07 b	3.42 ± 0.06 c	33.50 ± 0.87 d	10.17 ± 0.23 d	97.92 ± 0.88	29.73 ± 0.16	30.36 ± 0.11
*P* value							
Linear trend	0.315	0.369	0.387	0.420	0.442	0.114	0.281
Quadratic trend	0.037	< 0.0001	< 0.0001	< 0.0001	0.224	0.059	0.497

Mean ± standard error values followed by different letters in the same column are significantly different at *P* < 0.05

### Digestive enzymes activities

The digestive enzymes activities were substantially (*P* < 0.05) influenced by dietary NaCl, salinity stress, and their interaction (Table 4). The dietary NaCl linearly and quadratically (*P* < 0.05) improved mid-intestinal proteases, lipase, and α-amylase activities, and their highest values were detected in fish fed on 10 g NaCl/kg feed; after which, digestive enzyme activities were negatively declined. Moreover, salinity stress adversely (*P* < 0.05) affected digestive enzyme activities in all groups where their values were lower than those of before salinity stress (Table 4). It is noted that feeding fish on 10 g NaCl/kg feed restored the secretion of the digestive enzymes to be near the control group before salinity stress (Table 4).

**Table 4:** Activities of mid-intestinal digestive enzymes of Nile tilapia (*O. niloticus*) fed on different levels of sodium chloride (NaCl) for 60 days and further exposed to salinity stress (24 g/L)

Salinity stress	NaCl levels (g/kg feed)	Proteases (U/mg protein)	Lipase (U/mg protein)	α-Amylase (U/mg protein)
Individual treatment means ^a^				
Before salinity stress	0.00	20.22 ± 0.20 f	30.97 ± 0.74 de	26.77 ± 0.53 d
	5.0	33.95 ± 0.54 c	38.35 ± 0.01 b	34.88 ± 0.94 b
	10.0	39.99 ± 0.47 a	41.53 ± 0.67 a	40.52 ± 0.51 a
	15.0	36.78 ± 0.27 b	33.73 ± 2.01 c	35.34 ± 0.20 b
	20.0	30.00 ± 0.08 d	32.17 ± 0.27 cd	30.10 ± 0.24 c
After salinity stress	0.00	14.84 ± 1.44 h	17.77 ± 0.69 h	20.99 ± 1.20 e
	5.0	18.95 ± 0.23 fg	21.35 ± 0.38 g	16.96 ± 1.06 f
	10.0	23.75 ± 2.08 e	29.50 ± 0.44 e	29.36 ± 1.64 c
	15.0	18.48 ± 0.28 fg	24.68 ± 0.40 f	20.92 ± 0.26 e
	20.0	17.50 ± 0.30 g	21.72 ± 1.07 g	17.56 ± 0.58 f
Means of main effects ^b^				
Salinity stress				
Before stress	32.19	35.35	33.52	
After stress	18.70	23.00	21.16	
	Salt levels			
	0.0	17.53	24.37	23.88
	5.0	26.45	29.85	25.92
	10.0	31.87	35.52	34.94
	15.0	27.63	29.21	28.13
	20.0	23.75	26.95	23.83
Two-way ANOVA	*P* value			
Salinity stress	< 0.0001	< 0.0001	< 0.0001	
Dietary NaCl	< 0.0001	< 0.0001	< 0.0001	
Salinity stress × dietary NaCl	< 0.0001	0.002	< 0.0001	

^a^Treatment means represent the average values of three aquaria per treatment. Tukey HSD test was conducted for individual means only if there was a significant interaction (ANOVA, *P* < 0.05). Means in the same column followed by different letters are significantly different

^b^Main effect means in the same column followed by different letters are significantly different at *P* < 0.05 by Tukey HSD test

### Stress indices

As shown in Table 5, stress indices including glucose, T-CHO, AST, and ALT are markedly (*P* < 0.05) influenced by dietary NaCl, salinity stress, and their interaction but cortisol and TG are significantly (*P* < 0.05) affected by dietary NaCl and salinity stress only. With respect to the control treatment, cortisol, glucose, T-CHO, TG, AST, and ALT levels were significantly (*P* < 0.05) decreased with raising NaCl levels in fish diets up to 10 g NaCl/kg feed; after that level, marked increases in the previous parameters were observed (Table 5). On the other hand, during the salinity challenge, cortisol, glucose, T-CHO, TG, AST, and ALT levels were increased significantly (*P* < 0.05) compared with their values before salinity stress. Dietary NaCl had significantly (*P* < 0.05) restored the values of the parameters mentioned above to be near those before salinity stress (Table 5).

**Table 5:** Changes in blood cortisol, glucose, total cholesterol, triglycerides, aspartate aminotransferase (AST), and alanine transaminase (ALT) levels of Nile tilapia (*O. niloticus*) fed on different levels of sodium chloride (NaCl) for 60 days and further exposed to salinity stress (24 g/L)

Salinity stress	NaCl levels (g/kg feed)	Cortisol (ng/dL)	Glucose (mg/dL)	Cholesterol (mg/dL)	Triglyceride (mg/dL)	AST (U/L)	ALT (U/L)
Individual treatment means ^a^							
Before salinity stress	0.00	32.25 ± 1.22	122.10 ± 2.48 b	100.09 ± 0.14 bc	88.99 ± 0.51	33.08 ± 0.23 c	29.97 ± 0.05 c
	5.0	27.57 ± 1.08	104.13 ± 1.31 c	95.39 ± 0.51 cd	79.05 ± 0.46	30.13 ± 0.26 d	24.73 ± 1.30 d
	10.0	12.40 ± 0.62	87.03 ± 2.27 e	85.78 ± 0.33 f	68.51 ± 0.92	21.04 ± 0.36 f	15.59 ± 0.65 g
	15.0	17.73 ± 0.78	96.30 ± 1.50 cd	89.80 ± 0.25 ef	75.28 ± 1.34	25.73 ± 0.77 e	19.83 ± 0.18 f
	20.0	20.78 ± 0.89	101.73 ± 1.15 cd	90.72 ± 0.94 def	83.11 ± 1.46	28.51 ± 0.68 d	21.89 ± 0.39 e
After salinity stress	0.00	40.69 ± 0.38	163.50 ± 0.001 a	121.55 ± 4.79 a	93.50 ± 1.94	45.30 ± 0.74 a	40.15 ± 0.38 a
	5.0	35.57 ± 1.21	122.50 ± 6.07 b	105.35 ± 1.71 b	81.00 ± 0.77	40.80 ± 0.31 b	30.70 ± 0.32 c
	10.0	16.99 ± 1.07	95.70 ± 2.11 d	92.18 ± 1.39 de	73.17 ± 0.43	30.74 ± 1.39 cd	20.67 ± 1.01 ef
	15.0	22.98 ± 1.37	121.65 ± 2.22 b	97.85 ± 0.55 c	80.33 ± 0.59	32.74 ± 1.58 c	29.85 ± 0.68 c
	20.0	27.92 ± 1.28	128.40 ± 1.15 b	100.75 ± 0.29 bc	89.28 ± 0.73	41.23 ± 0.62 b	36.44 ± 0.31 b
Means of main effects ^b^							
Salinity stress							
Before stress	22.16 y	102.26	92.36	78.99 y	27.70	22.40	
After stress	28.83 x	126.35	103.54	83.46 x	38.16	31.56	
	Salt levels						
	0.0	36.47 a	142.80	110.82	91.25 a	39.19	35.06
	5.0	31.57 b	113.32	100.37	80.03 c	35.47	27.72
	10.0	14.70 e	91.37	88.98	70.84 e	25.89	18.13
	15.0	20.36 d	108.98	93.83	77.81 d	29.24	24.84
	20.0	24.35 c	115.07	95.74	86.20 b	34.87	29.17
Two-way ANOVA	*P* value						
Salinity stress	< 0.0001	< 0.0001	< 0.0001	< 0.0001	< 0.0001	< 0.0001	
Dietary NaCl	< 0.0001	< 0.0001	< 0.0001	< 0.0001	< 0.0001	< 0.0001	
Salinity stress × dietary NaCl	0.291	< 0.0001	0.002	0.373	0.018	< 0.0001	

^a^Treatment means represent the average values of three aquaria per treatment. Tukey HSD test was conducted for individual means only if there was a significant interaction (ANOVA, *P* < 0.05). Means in the same column followed by different letters are significantly different

^b^Main effect means in the same column followed by different letters (x and y for salinity stress and a–e for salt levels) are significantly different at *P* < 0.05 by Tukey HSD test

### The immune response

It is noted that total protein, albumin, and globulin were significantly (*P* < 0.05) influenced by dietary NaCl, salinity stress, and their interaction (Table 6). Before the salinity challenge, feeding Nile tilapia on dietary NaCl up to 10 g/kg feed showed highest levels of total protein, albumin, and globulin, but after this level, levels of those parameters decreased (Table 6). It is also noted that values of total protein, albumin, and globulin before salinity stress were higher than those found after salinity stress. Dietary NaCl significantly (*P* < 0.05) alleviated the salinity stress where total protein, albumin, and globulin values increased once again, especially in the fish group fed on a level of 10 g NaCl/kg diet (Table 6).

**Table 6:** Changes in the levels of blood total protein, albumin, and globulin of Nile tilapia (*O. niloticus*) fed on different levels of sodium chloride (NaCl) for 60 days and further exposed to salinity stress (24 g/L)

Salinity stress	NaCl levels (g/kg feed)	Total protein (g/L)	Albumin (g/L)	Globulin (g/L)
Individual treatment means ^a^				
Before salinity stress	0.00	3.75 ± 0.04 e	1.58 ± 0.07 d	2.17 ± 0.03 de
	5.0	4.95 ± 0.03 b	2.02 ± 0.02 b	2.93 ± 0.00 b
	10.0	6.09 ± 0.02 a	2.44 ± 0.04 a	3.65 ± 0.06 a
	15.0	4.53 ± 0.10 c	2.20 ± 0.05 b	2.33 ± 0.14 de
	20.0	3.85 ± 0.07 e	1.81 ± 0.10 c	2.04 ± 0.17 e
After salinity stress	0.00	2.71 ± 0.10 f	1.28 ± 0.01 e	1.43 ± 0.11 f
	5.0	3.90 ± 0.02 e	1.44 ± 0.09 de	2.46 ± 0.06 cd
	10.0	4.30 ± 0.13 d	1.63 ± 0.09 cd	2.67 ± 0.13 bc
	15.0	3.91 ± 0.05 e	1.59 ± 0.05 d	2.33 ± 0.01 de
	20.0	3.78 ± 0.05 e	1.35 ± 0.01 e	2.44 ± 0.07 cd
Means of main effects ^b^				
Salinity stress				
Before stress	4.63	2.01	2.62	
After stress	3.72	1.46	2.27	
	Salt levels			
	0.0	3.23	1.43	1.80
	5.0	4.43	1.73	2.70
	10.0	5.20	2.04	3.16
	15.0	4.22	1.90	2.33
	20.0	3.82	1.58	2.24
Two-way ANOVA	*P* value			
Salinity stress	< 0.0001	< 0.0001	< 0.0001	
Dietary NaCl	< 0.0001	< 0.0001	< 0.0001	
Salinity stress × dietary NaCl	< 0.0001	0.008	< 0.0001	

^a^Treatment means represent the average values of three aquaria per treatment. Tukey HSD test was conducted for individual means only if there was a significant interaction (ANOVA, *P* < 0.05). Means in the same column followed by different letters are significantly different

^b^Main effect means in the same column followed by different letters are significantly different at *P* < 0.05 by Tukey HSD test

Table 7 shows that dietary NaCl, salinity stress, and their interaction significantly (*P* < 0.05) affected lysozyme activity, phagocytic activity, and phagocytic index; meanwhile, RB activity and total Ig levels were affected by dietary NaCl and salinity stress only. The immune variables (lysozyme activity, RB activity, phagocytic activity, phagocytic index, and total Ig levels) were significantly (*P* < 0.05) enhanced by increasing dietary NaCl levels up to 10 g/kg feed, then they declined (Table 7). Subsequent to salinity stress, all immune parameters were significantly (*P* < 0.05) lower compared with values before salinity stress. Under salinity stress, feeding fish on 10 g NaCl/kg feed alleviated the salinity stress effects on immune biomarkers as compared with the control group exposed to salinity stress (Table 7).

**Table 7:** Changes in the levels of blood lysozyme, respiratory burst activity (RBA), phagocytic activity and index, and total immunoglobulin (total Ig) of Nile tilapia (*O. niloticus*) fed on different levels of sodium chloride (NaCl) for 60 days further exposed to salinity stress (24 g/L)

Salinity stress	NaCl levels (g/kg feed)	Lysozyme (µg/mL)	RBA (mg/mL)	Phagocytic activity (%)	Phagocytic index	Total Ig (mg/mL)
Individual treatment means ^a^						
Before salinity stress	0.00	6.61 ± 0.14 de	0.88 ± 0.07	8.81 ± 0.09 de	1.05 ± 0.02 cd	3.86 ± 0.06
	5.0	8.61 ± 0.15 c	1.14 ± 0.08	9.37 ± 0.22 cd	1.07 ± 0.02 cd	4.26 ± 0.04
	10.0	10.52 ± 0.15 a	1.48 ± 0.14	13.02 ± 0.36 a	1.30 ± 0.02 a	7.49 ± 0.32
	15.0	9.51 ± 0.03 b	1.04 ± 0.05	9.79 ± 0.30 c	1.01 ± 0.01 d	5.10 ± 0.04
	20.0	6.64 ± 0.39 de	0.97 ± 0.09	7.01 ± 0.37 f	1.21 ± 0.02 ab	4.87 ± 0.10
After salinity stress	0.00	4.30 ± 0.02 g	0.59 ± 0.02	8.02 ± 0.14 e	1.03 ± 0.02 d	3.24 ± 0.20
	5.0	6.57 ± 0.24 de	0.85 ± 0.05	8.48 ± 0.22 e	1.14 ± 0.02 bc	3.91 ± 0.08
	10.0	7.14 ± 0.06 d	1.25 ± 0.21	10.69 ± 0.26 b	1.24 ± 0.08 ab	6.43 ± 0.20
	15.0	6.14 ± 0.29 e	0.59 ± 0.05	8.83 ± 0.34 de	1.03 ± 0.04 d	4.74 ± 0.06
	20.0	5.04 ± 0.16 f	0.58 ± 0.02	8.11 ± 0.27 e	1.08 ± 0.05 cd	4.01 ± 0.19
Means of main effects ^b^						
Salinity stress						
Before stress	8.38	1.10 x	9.60	1.13	5.12 x	
After stress	5.84	0.77 y	8.83	1.10	4.47 y	
	Salt levels					
	0.0	5.46	0.74 d	8.42	1.04	3.55 d
	5.0	7.59	1.00 b	8.93	1.11	4.09 c
	10.0	8.83	1.37 a	11.86	1.27	6.96 a
	15.0	7.83	0.82 cd	9.31	1.02	4.92 b
	20.0	5.84	0.78 d	7.56	1.15	4.44 c
Two-way ANOVA	*P* value					
Salinity stress	< 0.0001	< 0.0001	< 0.0001	0.988	< 0.0001	
Dietary NaCl	< 0.0001	< 0.0001	< 0.0001	0.027	< 0.0001	
Salinity stress × dietary NaCl	< 0.0001	0.809	< 0.0001	< 0.0001	0.135	

^a^Treatment means represent the average values of three aquaria per treatment. Tukey HSD test was conducted for individual means only if there was a significant interaction (ANOVA, *P* < 0.05). Means in the same column followed by different letters are significantly different

^b^Main effect means in the same column followed by different letters (x and y for salinity stress and a–e for salt levels) are significantly different at *P* < 0.05 by Tukey HSD test

### Antioxidant activity and lipid peroxidation

As shown in Table 8, hepatic SOD and CAT activity were markedly (*P* < 0.05) influenced by dietary NaCl, salinity stress, and their interaction, but GPx and MDA levels were affected by dietary NaCl and salinity stress only. Before the salinity stress, hepatic SOD, CAT, and GPx levels were substantially (*P* < 0.05) improved with dietary NaCl, particularly at the level of 10 g/kg feed. Conversely, MDA levels were markedly (*P* < 0.05) decreased with increasing NaCl levels up to 10 g NaCl/kg feed, after which MDA values were significantly increased (Table 8). After salinity exposure, SOD, CAT, and GPx activities were significantly (*P* < 0.05) lower than their status before salinity stress; meanwhile, hepatic MDA values were substantially (*P* < 0.05) higher after salinity stress (Table 8). Feeding fish on dietary NaCl substantially restored the oxidative/antioxidant and lipid peroxidation status after salinity stress, particularly at the level of 10 g/kg feed (Table 8).

**Table 8:** Changes in hepatic superoxide dismutase (SOD), catalase (CAT), glutathione peroxidase (GPx), and malondialdehyde (MDA) levels of Nile tilapia (*O. niloticus*) fed on different levels of sodium chloride (NaCl) salt for 60 days and further exposed to salinity stress (24 g/L)

Salinity stress	NaCl levels (g/kg feed)	SOD (U/mg protein)	CAT (U/mg protein)	GPx (U/mg protein)	MDA (nmol/mg protein)
Individual treatment means ^a^					
Before salinity stress	0.00	10.13 ± 0.32 de	12.39 ± 0.04 e	17.61 ± 0.44	21.63 ± 0.94
	5.0	10.96 ± 0.70 cd	15.85 ± 0.86 c	20.81 ± 0.33	16.74 ± 0.85
	10.0	21.69 ± 1.17 a	27.57 ± 0.41 a	27.76 ± 1.11	13.73 ± 1.21
	15.0	12.35 ± 0.58 c	16.07 ± 0.11 c	19.95 ± 0.04	16.23 ± 0.21
	20.0	11.08 ± 0.34 cd	13.04 ± 0.40 de	14.79 ± 0.69	17.33 ± 0.32
After salinity stress	0.00	8.59 ± 0.55 e	10.96 ± 0.69 f	11.21 ± 0.44	27.13 ± 1.61
	5.0	10.54 ± 0.41 cde	14.33 ± 0.20 d	14.41 ± 0.33	22.29 ± 0.75
	10.0	17.39 ± 0.80 b	21.08 ± 0.54 b	21.36 ± 1.11	16.90 ± 1.28
	15.0	11.48 ± 0.68 cd	15.91 ± 0.54 c	13.55 ± 0.04	20.75 ± 0.42
	20.0	10.33 ± 0.26 cde	12.93 ± 0.08 de	8.39 ± 0.69	21.93 ± 2.16
Means of main effects ^b^					
Salinity stress					
Before stress	13.24	16.98	20.18 x	17.13 y	
After stress	11.67	15.04	13.78 y	21.8 x	
	Salt levels				
	0.0	9.36	11.68	14.41 c	24.38 a
	5.0	10.75	15.09	17.61 b	19.52 b
	10.0	19.54	24.33	24.56 a	15.32 c
	15.0	11.92	15.99	16.75 b	18.49 b
	20.0	10.71	12.99	11.59 d	19.63 b
Two-way ANOVA	*P* value				
Salinity stress	0.001	< 0.0001	< 0.0001	0.0001	
Dietary NaCl	< 0.0001	< 0.0001	< 0.0001	0.0001	
Salinity stress × dietary NaCl	0.040	< 0.0001	1.000	0.832	

^a^Treatment means represent the average values of three aquaria per treatment. Tukey HSD test was conducted for individual means only if there was a significant interaction (ANOVA, *P* < 0.05). Means in the same column followed by different letters are significantly different

^b^Main effect means in the same column followed by different letters (x and y for salinity stress and a–e for salt levels) are significantly different at *P* < 0.05 by Tukey HSD test

### Salinity challenge

**Fig. 2:**
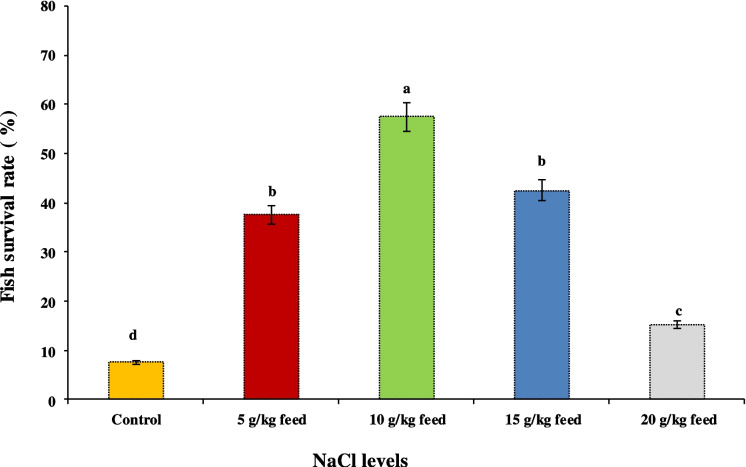
The survival rate (%) of Nile tilapia post-challenged with ascending salinity levels up to 32 g/L for 3 weeks after being fed on diets containing different dietary sodium chloride (NaCl) for 60 days. Bars assigned by different letters significantly differed at *P* < 0.05

**Fig. 3:**
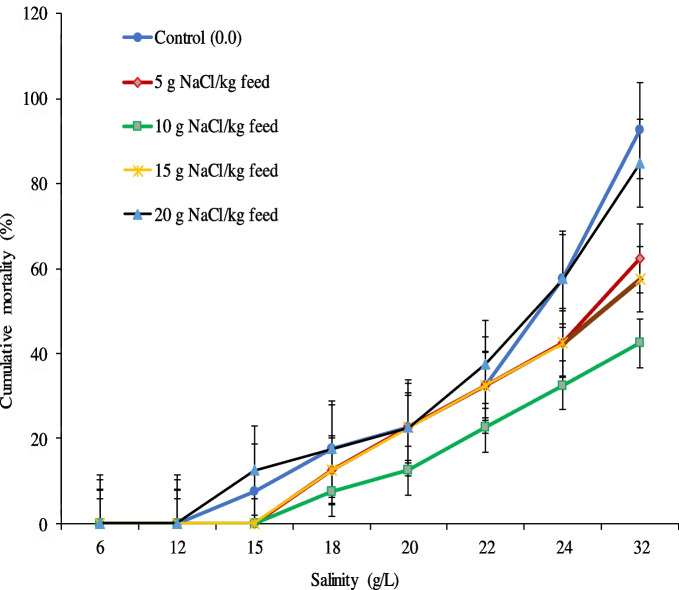
Cumulative mortality of Nile tilapia post-challenged with ascending salinity levels for 21 days after being fed on diets containing different NaCl levels for 60 days

Figure 2 shows the survivability, while Fig. 3 shows the cumulative mortality of Nile tilapia post-challenged with ascending salinity stress for 3 weeks after being fed on diets enriched with NaCl levels for 60 days. It was noticed that the highest fish survival (57.5%) after salinity exposure was found in fish fed on 10 g NaCl/kg feed; meanwhile, fish in the control group showed the lowest fish survivability (7.5%), followed by those fed on 20 g NaCl/kg feed (15.2%).

## Discussion

### The growth performance

In the present investigation, the highest growth indices of Nile tilapia were observed at the level of 10 g NaCl/kg feed. This was referred to as the significant enhancement in the feed intake that subsequently leads to enhanced fish growth. Additionally, feeding fish on NaCl resulted in marked enhancements in the secretion of intestinal digestive enzymes, which enhanced feed digestion and nutrient release and improved growth performance. In this regard, Yin et al. (2008) stated that dietary sodium chloride could enhance the fish’s appetite and promote nutrient absorption, which improves the fish’s growth. Previous studies evoked that dietary sodium chloride has been observed to enhance the growth and feed utilization in numerous fish species including Rohu carp, *Labeo rotiha* (Keshavanath et al. 2011), common carp, *Cyprinus carpio* (Nasir and Hamed 2016), shire tilapia, *O. shiranus* (Mzengereza and Kang’Ombe 2015), tambaqui, *Colossoma macropomum* (Keshavanath et al. 2012), and white grouper, *Epinephelus aeneus* (Cnaani et al. 2012). Fontaínhas-Fernandes et al. (2000) found that Nile tilapia showed higher growth performance when they were given a diet containing 8% NaCl for 3 months.

The reduction of growth performance of fish fed on 15 and 20 g NaCl/kg feed could be linked with lower feed intake and lower activities of digestive enzymes. In this context, Salman and Eddy (1988) stated that high levels of dietary NaCl caused low digestibility and faster food evacuation. MacLeod (1978) and Gangadhara et al. (2004) reported that excessive sodium chloride could adversely affect the feed intake. This is because high NaCl levels altered the gastric and intestinal environment and disrupted the digestion and absorption processes leading to growth reduction.

### Digestive enzymes activity

The present study noticed that a diet fortified with NaCl substantially enhanced the secretion and activity of digestive enzymes in comparison to the control treatment. This enhancement of digestive enzymes activity is consistent with what was found by Keshavanath et al. (2003), who found significant enhancements in proteases, α-amylase, and lipase levels in the intestine and hepatopancreas of Indian carps (*Labeo rohita*, *Cirrhinus mrigala*, and *C. carpio*) fed on diets enriched with 0.5–1.5% NaCl. Keshavanath et al. (2012) reported that dietary supplementation with 0.5–2.0% NaCl improved the digestibility of dry matter and protein in tambaqui, *C. macropomum*. Hallali et al. (2018) found that dietary supplementation of NaCl significantly increased the nutrient digestibility, peptide transporters expression, and modulated the gut microbial diversity that was associated with lipid digestibility.

It also noticed that salinity stress, in the current investigation, negatively affected the digestive enzymes secretion. In this regard, previous studies (Chen 1998; Yin et al. 2010) evoked that salinity inhibits enzymatic activities. These results could be attributed to variations in pH and ion concentration inside the digestive system resulted from the salt intake or the elimination of water during osmoregulatory processes (Nguyen et al. 2021). Tinh (2013) demonstrated that exposing snakehead fish (*Channa striata*) to 12 and 15 g/L salinity negatively affected the amylase activity in fish intestines. Conversely, feeding Nile tilapia on 10 g NaCl/kg feed minimized the adverse effects of salinity stress compared to those in the control fish group. This might be linked to the need to maintain homeostasis, which necessitates larger food consumption and elevated enzymatic activity (Usher et al. 1990; Bœuf and Payan 2001; Barman et al. 2005; Pujante et al. 2018).

### Hematological indices

The hematological analysis is one of the most critical techniques widely applied to assess fish’s health status and evaluate the circumstance-induced disease or stress that impacts fish productivity (Pavlidis et al. 2007). In the present investigation, dietary NaCl significantly improved Nile tilapia’s hematology, where RBCs, WBCs, Hct, and Hb levels were substantially higher in salt-fed fish, especially in the level of 10 g NaCl/kg feed. This improvement proves the essential function of dietary NaCl in improving fish health and welfare status without anemia symptoms. Furthermore, WBCs are often considered the primary immune cells, and it is worth noting that in fish, thrombocytes possess the capacity to contribute to the immune response via the production and release of several bioactive substances (Semple et al. 2011), as well as their ability to engage in phagocytosis (Stosik et al. 2001). Thus, in the present investigation, raising WBCs in fish fed on dietary NaCl indicates an increase in their innate immunity.

### Stress biomarkers

In this experiment, salinity stress increased blood cortisol and glucose levels, which indicates that fish have been stressed by salinity (Barton 2002; Pankhurst 2011). In addition, fish often release blood glucose as a means of getting energy to mitigate the adverse impacts of stress (Wendelaar Bonga 1997). In this regard, Laiz-Carrión et al. (2002) reported that fish exposed to elevated salinity levels had increased levels of cortisol and glucose in their bloodstream. As expected, in the current investigation, blood cortisol and glucose concentrations elevated substantially in all fish treatments subjected to salinity stress relative to their status before salinity stress. In a similar study, blood glucose and cortisol levels were shown to be considerably higher in tra catfish, *Pangasianodon hypophthalmus*, following their exposure to salinities of more than 12 g/L (Nguyen et al. 2014, 2021; Phuc et al. 2017). In other fish species, blood glucose and cortisol concentrations were also boosted in African catfish, *Clarias gariepinus* (Dawood et al. 2022b), and Nile tilapia, *O. niloticus* (Shukry et al. 2021) as well as yellowfin seabream (*Acanthopagrus latus*), and Asian seabass (*Lates calcarifer*) when subjected to elevated water salinities (Mozanzadeh et al. 2021).

On the one hand, blood cortisol and glucose contents decreased with raising dietary NaCl levels to 10 g/kg feed before and after salinity stress. This could be attributed to the oxidative breakdown of glucose, which is a critical process for providing energy to fish to cope with stress. Furthermore, it indicates that supplementing the diets with optimum NaCl levels can preserve energy that would be used for osmoregulation, leaving more energy accessible for metabolic patterns that deactivate the gluconeogenic pathway in addition to positive influences of NaCl in alleviating the negative impact of salinity stress.

Serum transaminases (AST and ALT) are considered as a biological mirror of the body’s health status and indicators of hepatic lesions and dysfunction (Sloss and Kubler 2009). In the current investigation, the rise in AST and ALT activities following salinity stress reflects the body’s responses to salinity stress and indicates damage, necrosis, and degeneration of hepatic tissues (Bruslé and Anadon 2017). In the current investigation, there are substantial reductions in serum AST and ALT levels in Nile tilapia-fed diets supplemented with NaCl, especially that fed on 10 g/kg feed, in comparison to the control treatment. Conversely, exposing the fish to salinity stress stimulated the secretion of AST and ALT, suggesting dysfunction and damage to liver tissues. Dawood et al. (2021) demonstrated that ALT activity was considerably higher in Nile tilapia that were exposed to 10 and 20 g/L salinity compared with the control treatment. Feeding fish on sodium chloride ameliorated the hepatic dysfunction induced by salinity stress. These consequences are compatible with assumptions by Huang et al. (2023), who concluded that dietary sodium chloride reduced hepatic dysfunction under cold stress.

### The innate immunity status

Blood protein profiles (TP, ALB, and GLOB) in the current study were higher in fish fed on NaCl-enriched diets, especially at the level of 10 g/kg feed. This could indicate the enhancements of fish’s humoral immunity (Patriche et al. 2009) as well as protein metabolism (Sardar et al. 2009). Additionally, the decrease in total protein after salinity stress in the current study might be explained as follows: during salinity stress, proteins undergo automatic degradation into amino acids and carbon compounds that are used as energy sources to cope with stress (Salahudeen 2004).

The respiratory burst (RB) activity refers to the rapid escalation in the generation of reactive oxygen species (ROS) that occurs during the process of phagocytosis of microorganisms. The RB activity plays a crucial role in innate immunity by facilitating the elimination of microorganisms by phagocytic cells (Secombes and Fletcher 1992). However, phagocytic cells play a crucial role in the cellular immune system of fish since they possess defensive mechanisms to combat attacking pathogens (Secombes and Fletcher 1992). Nevertheless, total Ig has a role in fish’s innate and adaptive immunity. One of its mechanisms of action is the triggering of complements, which opsonizes and lyses pathogens (Cooper 1985; Boshra et al. 2004). The immunoglobulins also facilitate pathogens clumping together for phagocytosis, elimination of pathogens, and destruction of cells (Ye et al. 2013). Likewise, lysozyme is essential for the breakdown of the cell walls of both pathogenic Gram-positive and Gram-negative bacteria (Ellis 1990; Saurabh and Sahoo 2008).

According to the present study, feeding Nile tilapia on NaCl-supplemented diets substantially increased lysozyme activity, RB activity, phagocytic capacity, and total Ig levels. These results indicate that dietary NaCl may have an immune-modulating influence on Nile tilapia. Moreover, salinity stress decreased the immune function of Nile tilapia, where the levels of above-mentioned immune indices significantly decreased. This outcome may be due to the disruption of osmoregulatory processes triggered by elevated salinity levels, as prior research indicates (Usha 2011; Soltanian et al. 2016). In similar studies, it is noted that heat stress and high stocking density stress have been shown to impair the Nile tilapia’s immune system (Dawood et al. 2020a, b).

### Antioxidant and lipid peroxidation activity

Antioxidant enzymes, such as SOD, CAT, and GPx, are the first line of protection from free radicals, especially reactive oxygen species (ROS) eliminating surplus reactive oxygen radicals, minimizing self-inflicted harm, and enhancing cellular functionality (Hossain et al. 2016; Abdel-Tawwab and Wafeek 2017; Hoseinifar et al. 2020). In improper salinity settings, the antioxidant system protects fish from environmental stress (Aliko et al. 2018). Salinity stress disrupts cell membrane biochemical equilibrium, creating many ROS (Dawood et al. 2021, 2023; Mozanzadeh et al. 2021; Moniruzzaman et al. 2022; Huang et al. 2023). Oxidative stress arises from a disparity between ROS and antioxidant capability, which may have negative effects on animals (Monaghan et al. 2009).

In this investigation, dietary NaCl significantly enhanced SOD, CAT, and GPx activities along with decreasing MDA. These consequences suggest that dietary NaCl showed antioxidant potentiality and may activate the antioxidant system in Nile tilapia (Moniruzzaman et al. 2022). In the current investigation, Nile tilapia fed with NaCl and exposed to salinity stress exhibited substantially lower hepatic MDA levels and higher hepatic SOD, CAT, and GPX activities than the control group. These data imply that feeding Nile tilapia on NaCl diets may boost salinity stress-exposed fish’s antioxidant capability.

The current research demonstrated that Nile tilapia subjected to salinity stress had reduced SOD, CAT, and GPX activities along with elevated MDA levels compared to their pre-salinity stress state. Reduced antioxidant enzymes activity may scavenge superoxide anions (Wang and Chen 2005). Alternatively, reduced SOD, CAT, and GPX activities may have produced higher lipid peroxidation indicator (MDA; Chaudière and Ferrari-Iliou 1999). The MDA levels are regarded as a biomarker of lipid peroxidation (LPO). The increase in MDA levels may result from ROS generation that oxidizes lipids (Del Rio et al. 2005). Thus, salinity stress might cause severe oxidative damage in Nile tilapia. Moniruzzaman et al. (2022) found that extreme salinity had a substantial influence on adult female *Notopterus chitala*, with MDA increasing and SOD and CAT decreasing at 12 g/L. Furthermore, published studies indicated that high and low salinity effects on freshwater fish might reduce or boost antioxidant enzyme activities (Dawood et al. 2022a, 2023; Moniruzzaman et al. 2022).

### Fish resistance to salinity stress

The majority of tilapia species can resist a broad range of salinity levels due to the fact that they are freshwater fish and are thought to have evolved from marine ancestors (Tavares-Dias 2022). The salinification of freshwater poses a significant risk to several fish species inhabiting freshwater ecosystems, as they may experience considerable physiological and osmotic stress, potentially leading to their vulnerability and endangerment (Jiang et al. 2022). Additionally, salinity exposure can diminish the pathogen tolerance of aquatic organisms (Zhang et al. 2016).

In the present study, feeding fish on NaCl levels for 60 days and further exposure gradually increased salinity levels up to 32 g/L over 3 weeks. Advantageous properties of dietary NaCl supplements on salinity tolerance were detected, especially in fish fed on a diet containing 10 g NaCl/kg feed, which showed higher fish survivability (57.5%). Moreover, fish-fed on the control diet (without salt feeding) or 20 g NaCl/kg feed showed much lower fish survivability (7.5% and 15.2%, respectively). This observation suggests that supplementing Nile tilapia with sodium chloride in their diets may enhance their resistance to salinity and mitigate the detrimental impacts of extremely high salinity levels via modulating osmoregulatory functions. This result is compatible with earlier short-term laboratory studies in which young black sea bass exhibited greater survival rates when given a diet containing 5 to 12.5% NaCl compared to those on a diet containing 0.0 to 2.5% NaCl when subjected to conditions of salt stress challenge (Alam et al. 2015).

Osmoregulation and metabolism may vary in later embryonic stages; juveniles, and adults that were exposed to varied ambient salinities, affecting fish survival and growth (Bœuf and Payan 2001; Takata et al. 2021). Diets supplemented with 10 g NaCl/kg feed also enhanced the survival rate through the transition to seawater of two tilapia species, *O. mossambicus* (Peters) and *O. spilurus* (Günther), as well as tilapia hybrid *O. aureus* x *O. niloticus* (Al-Amoudi 1987).

## Conclusion

The present investigation shows that feeding Nile tilapia on NaCl, particularly 10 g/kg feed, improved the growth performance and welfare status. In addition, dietary sodium chloride stimulates antioxidant and immune functions and inhibits the stress indices. Exposing the control fish group (free of dietary NaCl) to salinity stress also deteriorates the welfare status with immune suppression and elevated the stress indices. Meanwhile, feeding fish on a diet containing 10 g NaCl/kg feed could alleviate the adverse effects of salinity stress reducing fish mortality. Hence, this study recommends the addition of 7.5–10 g NaCl/kg feed to low-fishmeal diets for Nile tilapia. Further studies should be done to explore the mechanism and the osmoregulation channels of how dietary NaCl could minimize salinity stress and facilitate the possible culture of Nile tilapia in seawater.

## Data Availability

No datasets were generated or analysed during the current study.
